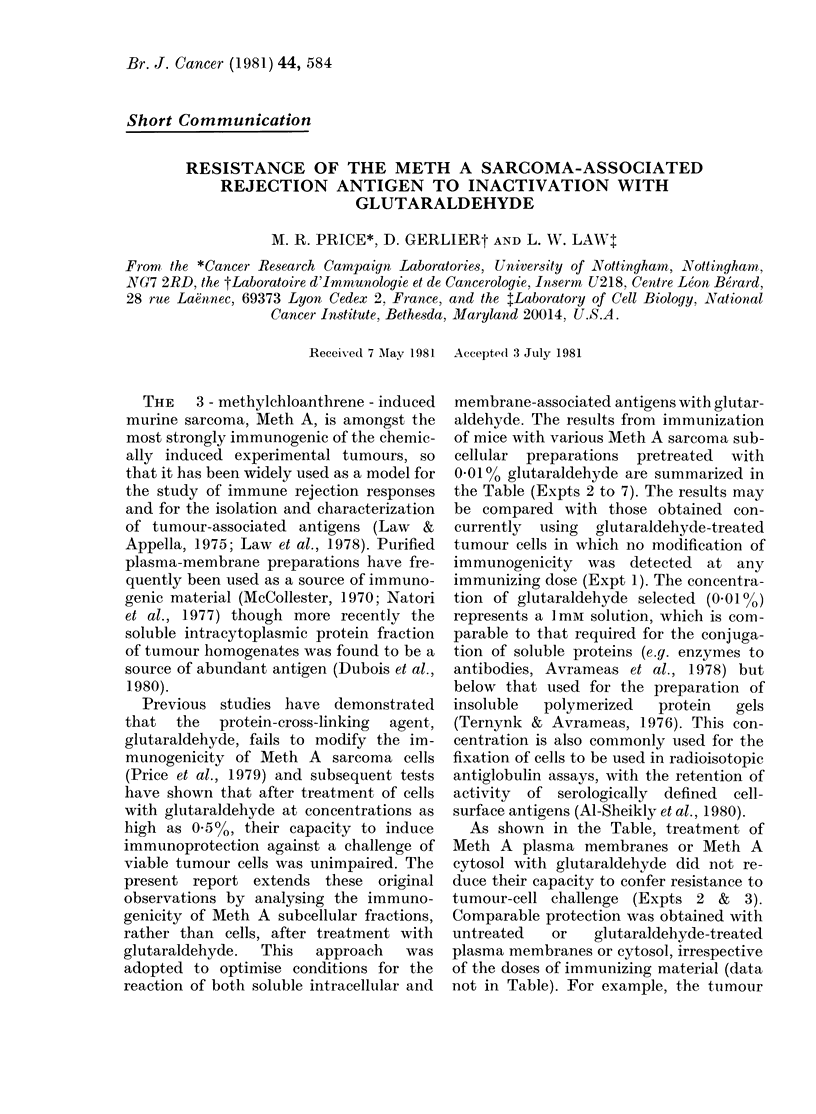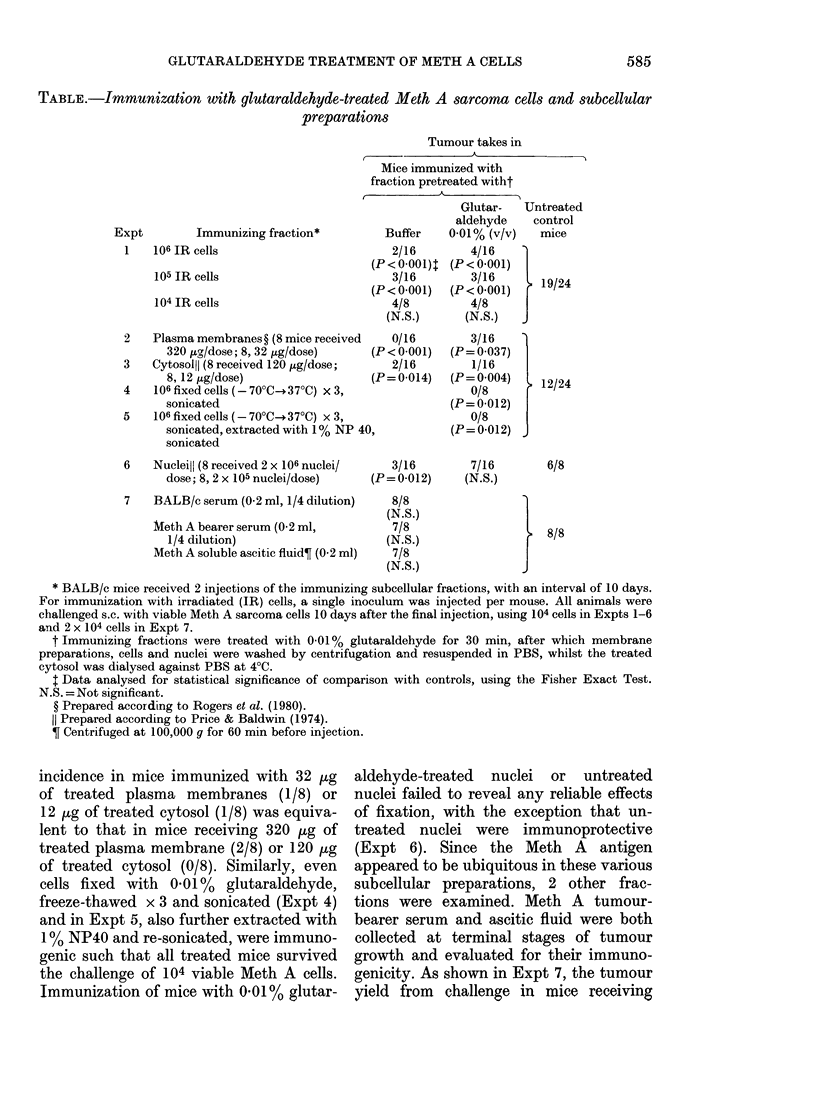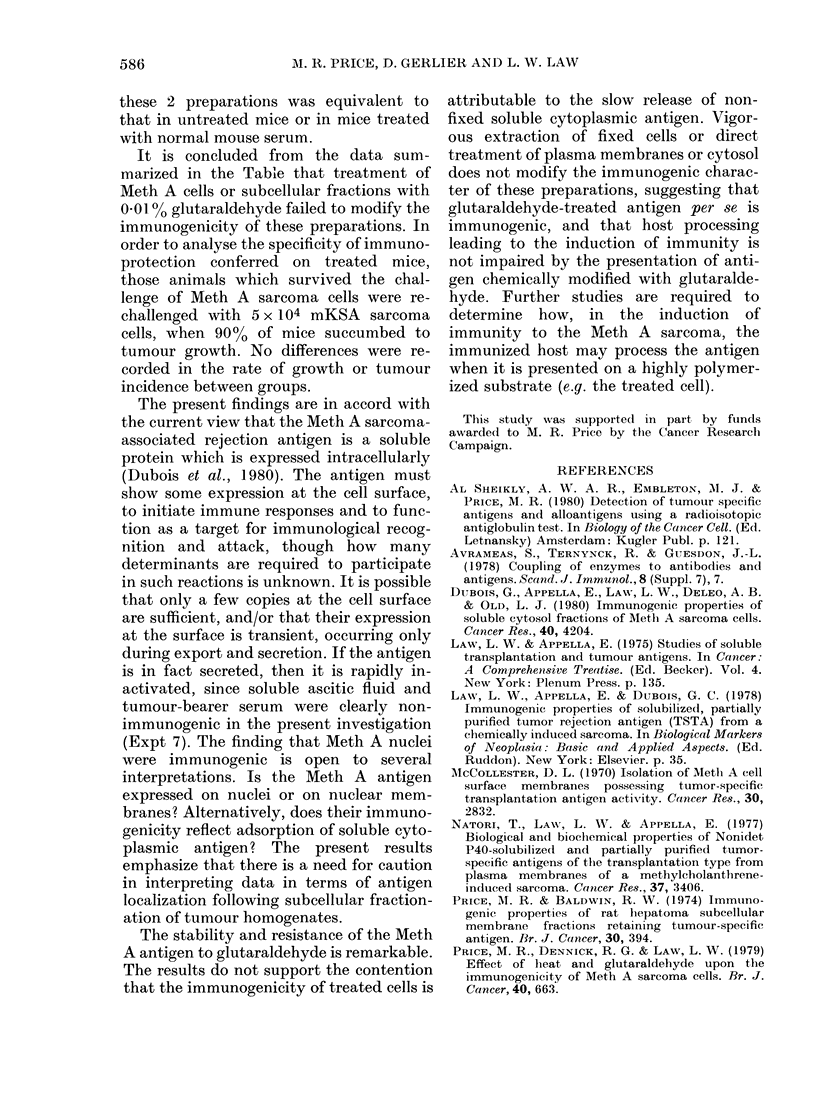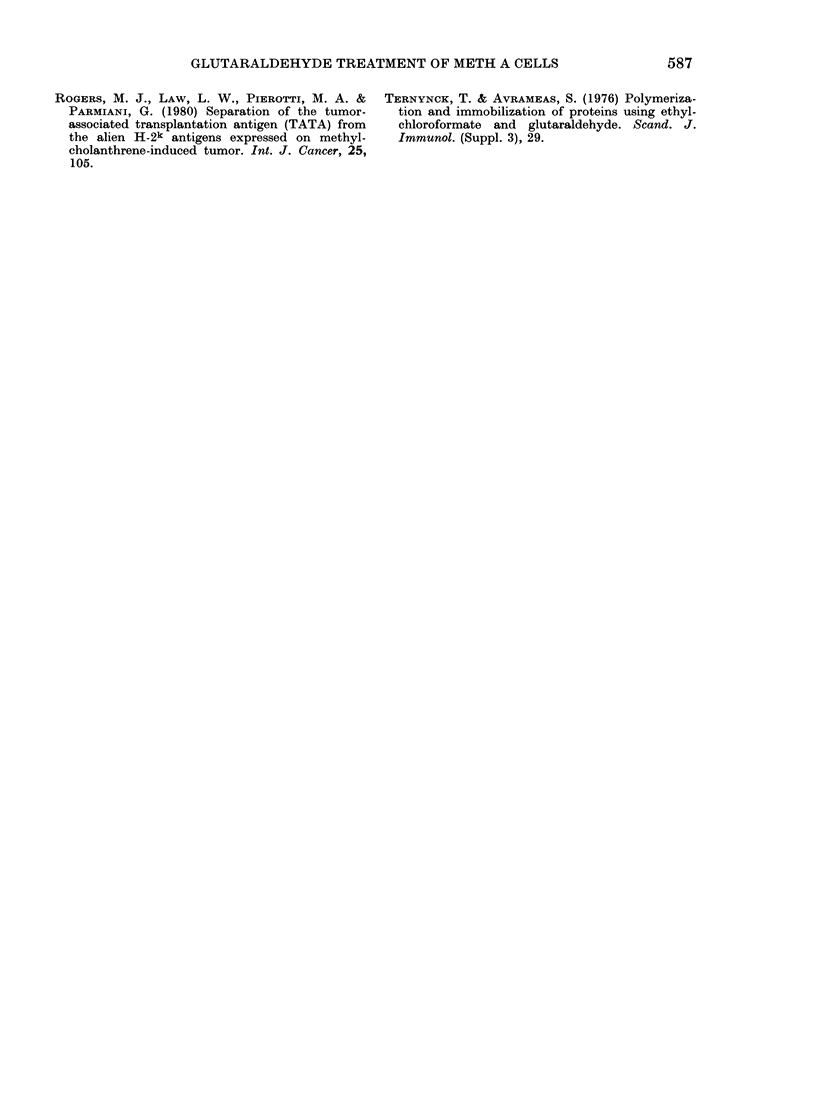# Resistance of the Meth A sarcoma-associated rejection antigen to inactivation with glutaraldehyde.

**DOI:** 10.1038/bjc.1981.230

**Published:** 1981-10

**Authors:** M. R. Price, D. Gerlier, L. W. Law


*Br. J. Cancer* (1981) **44**, 584

***Short Communication***

### RESISTANCE OF THE METH A SARCOMA-ASSOCIATED REJECTION ANTIGEN TO INACTIVATION WITH GLUTARALDEHYDE

M. R. PRICE*, D. GERLIER† AND L. W. LAW‡

*From the *Cancer Research Campaign Laboratories, University of Nottingham, Nottingham, NG7 2RD, the †Laboratoire d'Immunologie et de Cancerologie, Inserm U218, Centre Léon Bérard,* 28 *rue Laënnec,* 69373 *Lyon Cedex* 2, *France, and the ‡Laboratory of Cell Biology, National Cancer Institute, Bethesda, Maryland* 20014, *U.S.A.*

Received 7 May 1981 Accepted 3 July 1981

THE 3 - methylchloanthrene - induced murine sarcoma, Meth A, is amongst the most strongly immunogenic of the chemically induced experimental tumours, so that it has been widely used as a model for the study of immune rejection responses and for the isolation and characterization of tumour-associated antigens (Law & Appella, 1975; Law *et al.*, 1978). Purified plasma-membrane preparations have frequently been used as a source of immunogenic material (McCollester, 1970; Natori *et al.*, 1977) though more recently the soluble intracytoplasmic protein fraction of tumour homogenates was found to be a source of abundant antigen (Dubois *et al.*, 1980).

Previous studies have demonstrated that the protein-cross-linking agent, glutaraldehyde, fails to modify the immunogenicity of Meth A sarcoma cells (Price *et al.*, 1979) and subsequent tests have shown that after treatment of cells with glutaraldehyde at concentrations as high as 0·5%, their capacity to induce immunoprotection against a challenge of viable tumour cells was unimpaired. The present report extends these original observations by analysing the immunogenicity of Meth A subcellular fractions, rather than cells, after treatment with glutaraldehyde. This approach was adopted to optimise conditions for the reaction of both soluble intracellular and membrane-associated antigens with glutaraldehyde. The results from immunization of mice with various Meth A sarcoma subcellular preparations pretreated with 0·01% glutaraldehyde are summarized in the Table (Expts 2 to 7). The results may be compared with those obtained concurrently using glutaraldehyde-treated tumour cells in which no modification of immunogenicity was detected at any immunizing dose (Expt 1). The concentration of glutaraldehyde selected (0·01%) represents a 1mM solution, which is comparable to that required for the conjugation of soluble proteins (*e.g.* enzymes to antibodies, Avrameas *et al.*, 1978) but below that used for the preparation of insoluble polymerized protein gels (Ternynk & Avrameas, 1976). This concentration is also commonly used for the fixation of cells to be used in radioisotopic antiglobulin assays, with the retention of activity of serologically defined cell-surface antigens (Al-Sheikly *et al.*, 1980).

As shown in the Table, treatment of Meth A plasma membranes or Meth A cytosol with glutaraldehyde did not reduce their capacity to confer resistance to tumour-cell challenge (Expts 2 & 3). Comparable protection was obtained with untreated or glutaraldehyde-treated plasma membranes or cytosol, irrespective of the doses of immunizing material (data not in Table). For example, the tumour

TABLE.—*Immunization with glutaraldehyde-treated Meth A sarcoma cells and subcellular preparations*

| Expt | Immunizing fraction* | Tumour takes in: Mice immunized with fraction pretreated with† — Buffer | Glutaraldehyde 0·01% (v/v) | Untreated control mice |
|---|---|---|---|---|
| 1 | $10^6$ IR cells | 2/16 ($P<0·001$)‡ | 4/16 ($P<0·001$) | 19/24 |
| | $10^5$ IR cells | 3/16 ($P<0·001$) | 3/16 ($P<0·001$) | |
| | $10^4$ IR cells | 4/8 (N.S.) | 4/8 (N.S.) | |
| 2 | Plasma membranes§ (8 mice received 320 μg/dose; 8, 32 μg/dose) | 0/16 ($P<0·001$) | 3/16 ($P=0·037$) | 12/24 |
| 3 | Cytosol‖ (8 received 120 μg/dose; 8, 12 μg/dose) | 2/16 ($P=0·014$) | 1/16 ($P=0·004$) | |
| 4 | $10^6$ fixed cells (−70°C→37°C) × 3, sonicated | | 0/8 ($P=0·012$) | |
| 5 | $10^6$ fixed cells (−70°C→37°C) × 3, sonicated, extracted with 1% NP 40, sonicated | | 0/8 ($P=0·012$) | |
| 6 | Nuclei‖ (8 received 2 × $10^6$ nuclei/dose; 8, 2 × $10^5$ nuclei/dose) | 3/16 ($P=0·012$) | 7/16 (N.S.) | 6/8 |
| 7 | BALB/c serum (0·2 ml, 1/4 dilution) | 8/8 (N.S.) | | 8/8 |
| | Meth A bearer serum (0·2 ml, 1/4 dilution) | 7/8 (N.S.) | | |
| | Meth A soluble ascitic fluid¶ (0·2 ml) | 7/8 (N.S.) | | |

* BALB/c mice received 2 injections of the immunizing subcellular fractions, with an interval of 10 days. For immunization with irradiated (IR) cells, a single inoculum was injected per mouse. All animals were challenged s.c. with viable Meth A sarcoma cells 10 days after the final injection, using $10^4$ cells in Expts 1–6 and 2 × $10^4$ cells in Expt 7.

† Immunizing fractions were treated with 0·01% glutaraldehyde for 30 min, after which membrane preparations, cells and nuclei were washed by centrifugation and resuspended in PBS, whilst the treated cytosol was dialysed against PBS at 4°C.

‡ Data analysed for statistical significance of comparison with controls, using the Fisher Exact Test. N.S. = Not significant.

§ Prepared according to Rogers *et al.* (1980).

‖ Prepared according to Price & Baldwin (1974).

¶ Centrifuged at 100,000 *g* for 60 min before injection.

incidence in mice immunized with 32 μg of treated plasma membranes (1/8) or 12 μg of treated cytosol (1/8) was equivalent to that in mice receiving 320 μg of treated plasma membrane (2/8) or 120 μg of treated cytosol (0/8). Similarly, even cells fixed with 0·01% glutaraldehyde, freeze-thawed × 3 and sonicated (Expt 4) and in Expt 5, also further extracted with 1% NP40 and re-sonicated, were immunogenic such that all treated mice survived the challenge of $10^4$ viable Meth A cells. Immunization of mice with 0·01% glutaraldehyde-treated nuclei or untreated nuclei failed to reveal any reliable effects of fixation, with the exception that untreated nuclei were immunoprotective (Expt 6). Since the Meth A antigen appeared to be ubiquitous in these various subcellular preparations, 2 other fractions were examined. Meth A tumour-bearer serum and ascitic fluid were both collected at terminal stages of tumour growth and evaluated for their immunogenicity. As shown in Expt 7, the tumour yield from challenge in mice receiving these 2 preparations was equivalent to that in untreated mice or in mice treated with normal mouse serum.

It is concluded from the data summarized in the Table that treatment of Meth A cells or subcellular fractions with 0·01% glutaraldehyde failed to modify the immunogenicity of these preparations. In order to analyse the specificity of immunoprotection conferred on treated mice, those animals which survived the challenge of Meth A sarcoma cells were rechallenged with $5 \times 10^4$ mKSA sarcoma cells, when 90% of mice succumbed to tumour growth. No differences were recorded in the rate of growth or tumour incidence between groups.

The present findings are in accord with the current view that the Meth A sarcoma-associated rejection antigen is a soluble protein which is expressed intracellularly (Dubois *et al.*, 1980). The antigen must show some expression at the cell surface, to initiate immune responses and to function as a target for immunological recognition and attack, though how many determinants are required to participate in such reactions is unknown. It is possible that only a few copies at the cell surface are sufficient, and/or that their expression at the surface is transient, occurring only during export and secretion. If the antigen is in fact secreted, then it is rapidly inactivated, since soluble ascitic fluid and tumour-bearer serum were clearly non-immunogenic in the present investigation (Expt 7). The finding that Meth A nuclei were immunogenic is open to several interpretations. Is the Meth A antigen expressed on nuclei or on nuclear membranes? Alternatively, does their immunogenicity reflect adsorption of soluble cytoplasmic antigen? The present results emphasize that there is a need for caution in interpreting data in terms of antigen localization following subcellular fractionation of tumour homogenates.

The stability and resistance of the Meth A antigen to glutaraldehyde is remarkable. The results do not support the contention that the immunogenicity of treated cells is attributable to the slow release of non-fixed soluble cytoplasmic antigen. Vigorous extraction of fixed cells or direct treatment of plasma membranes or cytosol does not modify the immunogenic character of these preparations, suggesting that glutaraldehyde-treated antigen *per se* is immunogenic, and that host processing leading to the induction of immunity is not impaired by the presentation of antigen chemically modified with glutaraldehyde. Further studies are required to determine how, in the induction of immunity to the Meth A sarcoma, the immunized host may process the antigen when it is presented on a highly polymerized substrate (*e.g.* the treated cell).

This study was supported in part by funds awarded to M. R. Price by the Cancer Research Campaign.

REFERENCES

Al Sheikly, A. W. A. R., Embleton, M. J. & Price, M. R. (1980) Detection of tumour specific antigens and alloantigens using a radioisotopic antiglobulin test. In *Biology of the Cancer Cell.* (Ed. Letnansky) Amsterdam: Kugler Publ. p. 121.

Avrameas, S., Ternynck, R. & Guesdon, J.-L. (1978) Coupling of enzymes to antibodies and antigens. *Scand. J. Immunol.*, **8** (Suppl. 7), 7.

Dubois, G., Appella, E., Law, L. W., Deleo, A. B. & Old, L. J. (1980) Immunogenic properties of soluble cytosol fractions of Meth A sarcoma cells. *Cancer Res.*, **40**, 4204.

Law, L. W. & Appella, E. (1975) Studies of soluble transplantation and tumour antigens. In *Cancer: A Comprehensive Treatise.* (Ed. Becker). Vol. 4. New York: Plenum Press. p. 135.

Law, L. W., Appella, E. & Dubois, G. C. (1978) Immunogenic properties of solubilized, partially purified tumor rejection antigen (TSTA) from a chemically induced sarcoma. In *Biological Markers of Neoplasia: Basic and Applied Aspects.* (Ed. Ruddon). New York: Elsevier. p. 35.

McCollester, D. L. (1970) Isolation of Meth A cell surface membranes possessing tumor-specific transplantation antigen activity. *Cancer Res.*, **30**, 2832.

Natori, T., Law, L. W. & Appella, E. (1977) Biological and biochemical properties of Nonidet P40-solubilized and partially purified tumor-specific antigens of the transplantation type from plasma membranes of a methylcholanthrene-induced sarcoma. *Cancer Res.*, **37**, 3406.

Price, M. R. & Baldwin, R. W. (1974) Immunogenic properties of rat hepatoma subcellular membrane fractions retaining tumour-specific antigen. *Br. J. Cancer*, **30**, 394.

Price, M. R., Dennick, R. G. & Law, L. W. (1979) Effect of heat and glutaraldehyde upon the immunogenicity of Meth A sarcoma cells. *Br. J. Cancer*, **40**, 663.

ROGERS, M. J., LAW, L. W., PIEROTTI, M. A. & PARMIANI, G. (1980) Separation of the tumor-associated transplantation antigen (TATA) from the alien H-$2^k$ antigens expressed on methyl-cholanthrene-induced tumor. *Int. J. Cancer*, **25**, 105.

TERNYNCK, T. & AVRAMEAS, S. (1976) Polymerization and immobilization of proteins using ethyl-chloroformate and glutaraldehyde. *Scand. J. Immunol.* (Suppl. 3), 29.